# A Compact and Low Power RO PUF with High Resilience to the EM Side-Channel Attack and the SVM Modelling Attack of Wireless Sensor Networks

**DOI:** 10.3390/s18020322

**Published:** 2018-01-23

**Authors:** Yuan Cao, Xiaojin Zhao, Wenbin Ye, Qingbang Han, Xiaofang Pan

**Affiliations:** 1 College of Internet of Things Engineering, Hohai University, Changzhou 213022, China; 20161965@hhu.edu.cn (Y.C.); 20111841@hhu.edu.cn (Q.H.); 2 College of Electronic Science and Technology, Shenzhen University, Shenzhen 518060, China; yewenbin@szu.edu.cn; 3 College of Information Engineering, Shenzhen University, Shenzhen 518060, China; eexpan@szu.edu.cn

**Keywords:** wireless sensor network, strong physical unclonable function, ring oscillator, electromagnetic side-channel attack, support vector machine modelling attack

## Abstract

Authentication is a crucial security service for the wireless sensor networks (WSNs) in versatile domains. The deployment of WSN devices in the untrusted open environment and the resource-constrained nature make the on-chip authentication an open challenge. The strong physical unclonable function (PUF) came in handy as light-weight authentication security primitive. In this paper, we present the first ring oscillator (RO) based strong physical unclonable function (PUF) with high resilience to both the electromagnetic (EM) side-channel attack and the support vector machine (SVM) modelling attack. By employing an RO based PUF architecture with the current starved inverter as the delay cell, the oscillation power is significantly reduced to minimize the emitted EM signal, leading to greatly enhanced immunity to the EM side-channel analysis attack. In addition, featuring superior reconfigurability due to the conspicuously simplified circuitries, the proposed implementation is capable of withstanding the SVM modelling attack by generating and comparing a large number of RO frequency pairs. The reported experimental results validate the prototype of a 9-stage RO PUF fabricated using standard 65 nm complementary-metal-oxide-semiconductor (CMOS) process. Operating at the supply voltage of 1.2 V and the frequency of 100 KHz, the fabricated RO PUF occupies a compact silicon area of 250 μm${}^{2}$ and consumes a power as low as 5.16 μW per challenge-response pair (CRP). Furthermore, the uniqueness and the worst-case reliability are measured to be 50.17% and 98.30% for the working temperature range of −40∼120 ${}^{\circ }$C and the supply voltage variation of ±2%, respectively. Thus, the proposed PUF is applicable for the low power, low cost and secure WSN communications.

## 1. Introduction

Wireless sensor networks (WSNs) are intensely ubiquitous and encompass a broad range of new applications, such as AR/VR, Internet of Things (IoT), and vehicle network [1]. The smart sensors are the tentacles that sense the environmental conditions and communicate the information that is usually security and private critical. It is paramount to assure the integrity of the sensing and protect the collected information from malicious attacks during the data transmission through the untrusted communication channels. However, it is not feasible to implement the conventional cryptography modules on the resource-constrained sensor nodes, including advanced encryption standard (AES), elliptic curve cryptography (ECC) and so on. In light of this, the physical unclonable function (PUF) as a lightweight security primitive has raised more and more interests in the WSN research community [2,3].

PUF, broadly categorized as “weak PUF” and “strong PUF”, is an emerging low-cost on-chip secure primitive [2,4,5,6,7]. By converting the inherent uncontrollable and unpredictable variations of the semiconductor manufacturing process to a random digital bit string, a PUF generates a “response” upon a “challenge”, namely a challenge-response pair (CRP). Weak PUFs target the application of the key generation [2,4], while strong PUFs are more suitable for the device authentication [8]. The weak PUFs generally have very few CRPs. They can be essentially regarded as a special form of memory, which are resilient to the invasive attacks [8]. The strong PUF, as a disorderd physical system, features complex challenge-response behavior and large challenge-response space. It is almost impossible to physically clone a strong PUF, whose CRPs behave exactly the same as the original one. As a result, the strong PUF is more suitable for the wide range of device authentication applications than its counterpart of weak PUF [9,10,11]. Among the strong PUF implementations, ring oscillator (RO) PUF is superior due to the following reasons [12]: (1) the frequencies of the ROs in the PUF are irrelevant to the delay introduced by the outputs’ routing; (2) the difference of the RO pairs’ frequencies can be further increased by extending their oscillation time. However, the feature of public CRPs’ accessability for strong PUFs renders them vulnerable to modeling attacks [8]. In addition, Merli’s work demonstrates that the electromagnetic (EM) measurements are capable of disclosing both the frequency and the location of each RO, which enables the prediction of the RO PUF’s CRPs [12].

To address these security threats, this paper presents the first RO based strong PUF design to resist the EM side-channel attack and the support vector machine (SVM) modelling attack. Compared with the previous implementations, the proposed RO PUF features low power consumption, high area efficiency and improved security performance with high entropy. Specifically, in order to minimize the influence of the emitted EM signal, the RO’s delay cell consists of the current starved inverters, which operate at the subthreshold region and significantly reduce the overall power consumption. In addition, each RO used to generate the CRP includes one of the two current starved inverters in each inverter stage, resulting in the exponential increment of the RO number in a given area. Furthermore, the RO PUF architecture employs a linear feedback shift register (LFSR) counter to enhance the system’s logical reconfigurability and thwart the SVM modelling attack. The rest of the paper is organized as follows. Section 2 introduces the architecture and operation of proposed RO PUF. Experiments results are presented and discussed in Section 3. Finally, the conclusion is drawn in Section 4.

## 2. Architecture and Operation of Proposed RO PUF

**Figure 1:**
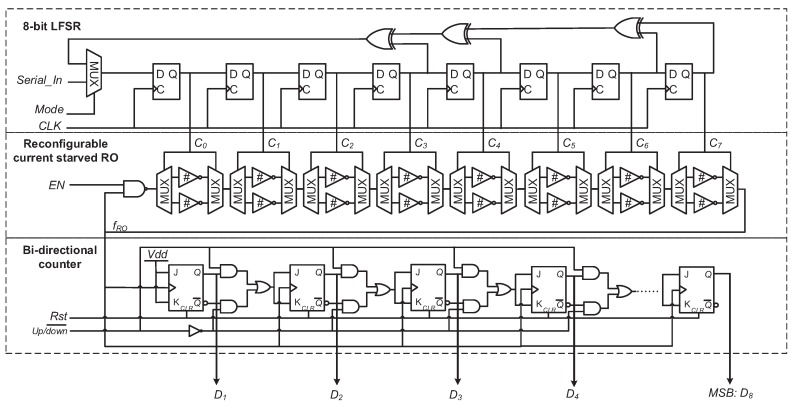
Architecture of the proposed ring oscillator (RO) PUF.

**Figure 2:**
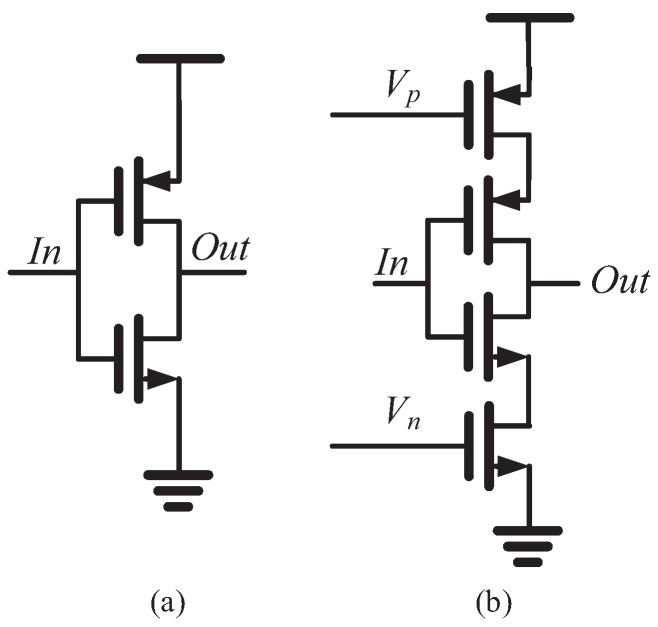
Schematics of (**a**) regular inverter and (**b**) current starved inverter.

Figure 1 illustrates the architecture of the proposed 9-stage RO PUF. It basically consists of one LFSR counter, one reconfigurable current starved RO and one bidirectional counter. The key component is the reconfigurable current starved RO. Its oscillation frequency is determined by the delay $t_{d}$ of its current starved inverter stage. Here $t_{d}$ is given as:

(1) $$t_{d}=\frac{C_{0}V_{dd}}{i_{D}}$$

where $C_{0}$ is the load capacitance, $i_{D}$ is the average charging/discharging current and $V_{dd}$ is the power supply voltage. The key component in the proposed RO PUF is the MUX based current starved RO with reconfigurability. The delay cell in the RO is the current starved inverter. As shown in Figure 2, two additional bias transistors are added into the regular inverter to realize the current starved inverter.

**Figure 3:**
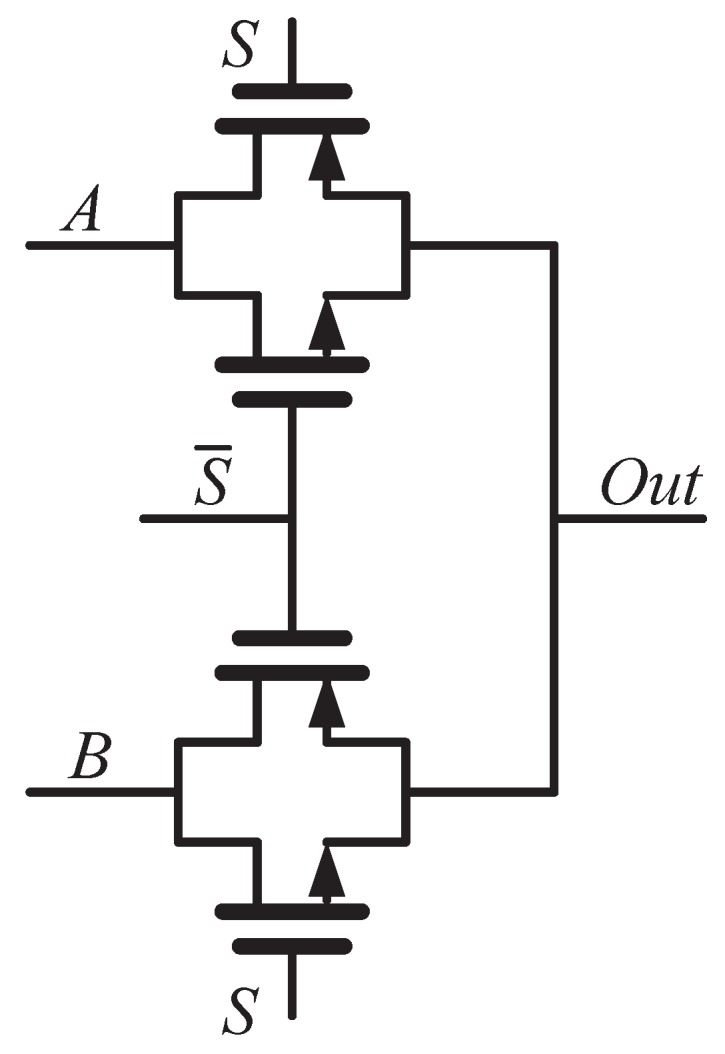
Schematic of the MUX in the proposed physical unclonable function (PUF).

In the current starved inverter, the output capacitance is charged/discharged by the maximum drain current $I_{D}$ of the pull-up/pull-down transistor initially, which is decreased during the transition. Without considering the leakage and short-circuit current, the average current $i_{D}$ is equal to $\eta I_{D}$, where the fraction η is fixed for a given device. The current starved inverter can be made to work in the sub-threshold region by biasing the voltages $V_{p}$ and $V_{n}$. The maximum current $I_{D}$ is formulated by [13]:

(2) $$I_{D}=\mu C_{OX}\frac{W}{L}{\left(\frac{\kappa_{B}T}{q}\right)}^{2}\left(n-1\right)e^{\frac{q(V_{GS}-V_{t})}{n\kappa_{B}T}}\left(1-e^{-\frac{qV_{D}}{\kappa_{B}T}}\right)$$

(3) $$n=\frac{1+(C_{S}+C_{it})}{C_{OX}}$$

where $\kappa_{B}$ is the Boltzmann constant; $C_{S}$, $C_{it}$ and $C_{ox}$ are the capacitances associated with the semiconductor, fast surface states and gate oxide, respectively. From (2), the current is controlled by the $V_{GS}$ of each transistor. Therefore, the power and oscillation frequency of the RO can be tuned by $V_{p}$ and $V_{n}$. It should be noted that two multiplexors are placed in each inverter stage: one at the gate outputs, the other one at the gate inputs. The multiplexors are realized with transmission gates to reduce their delay and transistor count as shown in Figure 3. It is difficult to model the temperature dependency of the multiplexors as the transistors can operate in several regions [14]. What is more feasible is to increase their transistors’ width to make their contribution to the timing variation of the RO negligible relative to the inverters.

Each response bit of this PUF is produced by comparing two randomly selected ROs’ frequencies. The operation of the proposed PUF is explained as follows. The LFSR counter is firstly fed with an 8-bit challenge *C* through Serial_In port by setting the signal Mode high. EN in the RO is set to low to disable the oscillation. After $C^{\prime }$ from the LFSR counter settles down, EN is set to high. Then a rising edge of Rst is applied to reset the bidirectional counter, which can record the RO’s output frequency. Suppose the RO selected by *C* is $RO_{A}$. Its oscillation frequency $f_{A}$ is measured by the bidirectional counter with $Up/\bar{down}$ equal to high for a specific time of *t*. Similarly, another challenge $C^{\prime }$ is generated by shifting *C* in the LFSR counter with $N_{clk}$ ($N_{clk}<2^{8}$) cycles. The frequency $f_{B}$ of the new RO $RO_{B}$ is recorded by the bidirectional counter with $Up/\bar{down}$ set to low for the time of *t*. Finally, the recorded value in the bidirectional counter represents the frequency difference of the RO pair (i.e. $RO_{A}$ and $RO_{B}$). The most significant bit (MSB) of the bidirectional counter is used as the PUF’s output bit. In the proposed RO PUF, the heat generated by the oscillation of $RO_{A}$ is too small and can be negligible due to the low power feature of the current starved inverter working in the subthreshold region.

It is noted that, with a different $N_{clk}$ in the LFSR counter, the same input challenge can produce different responses for the same PUF instance. As a result, this structure can be considered as a logically reconfigurable PUF [15], In contrast to the controlled PUF (CPUF) [16], which adds simple hash functions to a PUF to forbid the untrusted user to access the PUF directly, the proposed PUF allows for changing the CRP behavior by varying $N_{clk}$, without the physical replacement and modification of the underlying PUF. This logical reconfigurability makes the proposed PUF more resilient to SVM based attack, which is a subclass of modeling attacks. Modeling attack assumes that the adversary can create a model of the targeted PUF, given a number of CRPs [8]. The rest CRPs can be predicted with the help of this model. To predict the CRPs with SVM, the attackers need to model the CRP generation accurately. Without considering the reconfigurability, $N_{clk}$ for the LFSR counter is assumed to be a known constant. The oscillation frequency of each RO in the proposed PUF is reversely proportional to the sum of the delay in each stage. The applied challenge $C/C^{\prime }$ selects the top or bottom path. An additive delay model for the proposed PUF structure can be constructed as follows. The response corresponding to the challenge *C* can be expressed as:

(4) $$\begin{array}{c} R=\left\{\begin{array}{ccc} 1 & \mathrm{if} & \delta \left(n+1\right)>\delta^{\prime }\left(n+1\right)\\ -1 & \mathrm{if} & \delta \left(n+1\right)<\delta^{\prime }\left(n+1\right) \end{array}\right. \end{array}$$

where δ(n+1) and $\delta^{\prime }\left(n+1\right)$ are the signal delays from the NAND gate input to the output of the RO’s last inverter stage (i.e., the (n+1)-th) in Figure 1, upon the application of the challenge *C* and the shadow challenge $C^{\prime }$ after $N_{clk}$ cycles, respectively. These two delays are then written as:

(5) $$\begin{array}{ccc} \delta (i) & = & \frac{1+C_{i}}{2}p_{i}+\frac{1-C_{i}}{2}q_{i}+\delta \left(i-1\right) \end{array}$$

(6) $$\begin{array}{ccc} \delta^{\prime }\left(i\right) & = & \frac{1+C_{i}^{\prime }}{2}p_{i}+\frac{1-C_{i}^{\prime }}{2}q_{i}+\delta^{\prime }\left(i-1\right) \end{array}$$

where $p_{i}$ and $q_{i}$ (i=1,2,⋯,n+1) are the top and bottom inverter delays at the *i*-th inverter stage of the RO, respectively, and $C_{i},C_{i}^{\prime }\in \left\{-1,1\right\}$. In contrast to the arbiter PUF whose two competing-signals are generated at the same time (by the rising edge from the very beginning of the delay chain), the proposed RO PUF generates the two competing-signals at different times (one is generated when Challenge *C* is applied, the other one is generated when the shadow Challenge $C^{\prime }$ is applied). The frequency distance between two selected ROs is then calculated by subtraction with the help of the following bi-directional counter. Let Δ(i) denote the difference between δ(i) and $\delta^{\prime }\left(i\right)$. By subtracting (5) from (6), we can have:

(7) $$\begin{array}{ccc} \Delta (i) & = & \frac{p_{i}-q_{i}}{2}\left(C_{i}-C_{i}^{\prime }\right)+\Delta \left(i-1\right) \end{array}$$

(8) $$\begin{array}{ccc} \Delta (i) & = & \frac{p_{i}-q_{i}}{2}\left(C_{i}-C_{i}^{\prime }\right)+\\ & & \frac{p_{i-1}-q_{i-1}}{2}\left(C_{i-1}-C_{i-1}^{\prime }\right)\cdots +\Delta \left(0\right) \end{array}$$

where Δ(0)=0. The final delay difference Δ(n+1) can be represented as an inner product:

(9) $$\Delta \left(n+1\right)=<\vec{w},\vec{x}>$$

where $\vec{w}=\frac{1}{2}\left(\left(p_{0}-q_{0}\right),\cdots ,\left(p_{n+1}-q_{n+1}\right)\right)$ and $\vec{x}=\left(\left(C_{0}-C_{0}^{\prime }\right),\cdots ,\left(C_{n+1}-C_{n+1}^{\prime }\right)\right)$. In this way, a separating hyperplane in the space of all feature vectors $\vec{x}$ can be determined by the SVM. However, if $N_{clk}$ is not fixed but randomly reconfigurable by the user, $\vec{x}$ becomes unpredictable. In order to secure $N_{clk}$, $N_{clk}$ can be encrypted and sent along with the challenge to the device when an authentication is inquired.

## 3. Experimental Results and Discussions

**Figure 4:**
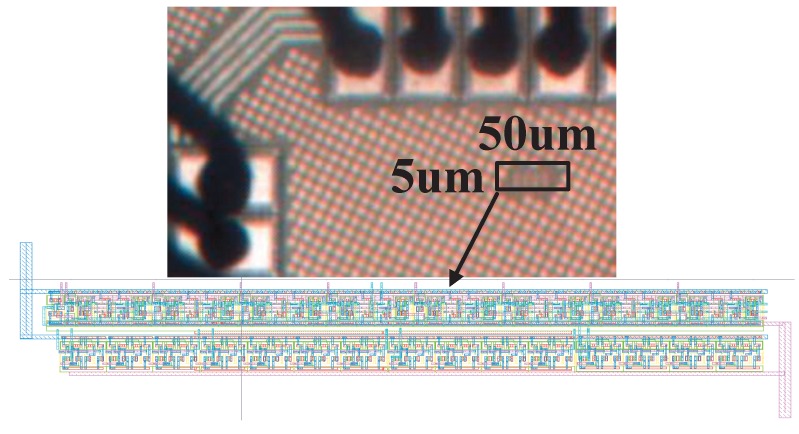
The microphotograph of the proposed RO PUF chip.

**Figure 5:**
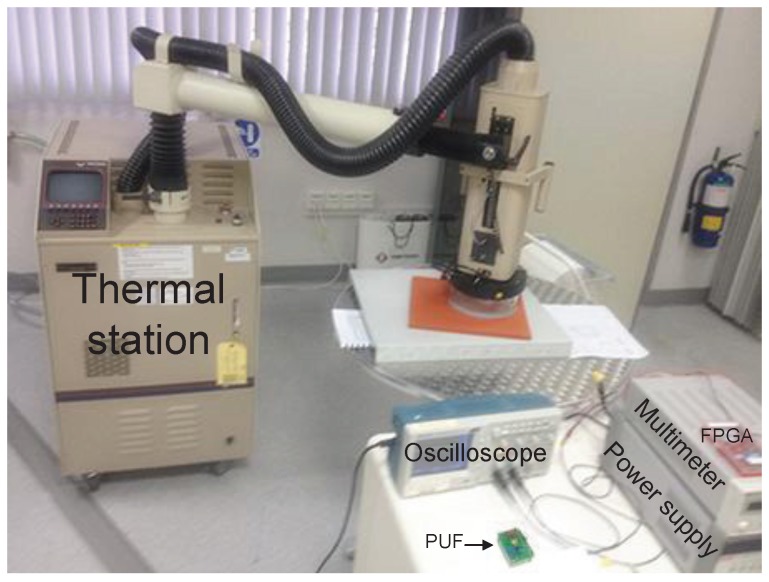
The probe station for testing the sample chips.

**Figure 6:**
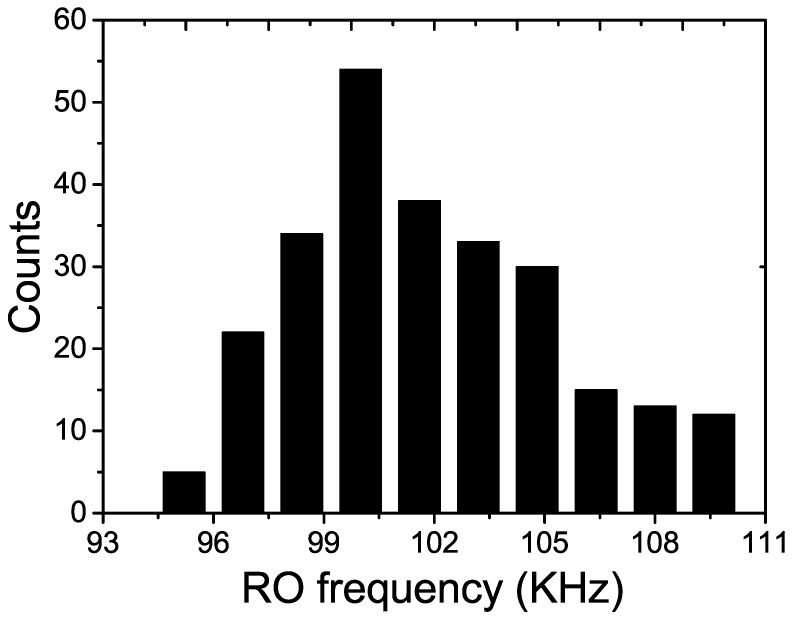
The distribution of RO’s oscillation frequency for one sample chip.

The microphotograph of the fabricated chip using standard 65 nm CMOS process is shown in Figure 4. The proposed PUF’s core area is only 5 × 50 μm${}^{2}$. The setup of the probe station for the post-silicon test is shown in Figure 5, where *Agilent* oscilloscope with 1GS/s sampling rate is used to measure the output frequencies of the current starved ROs and capture the responses of the PUF. The LFSR counter and other control signals are generated by a *Xilinx Virtex-II Pro* FPGA board externally. Figure 6 shows the distribution of RO’s oscillation frequency in one sample chip. There are totally 2${}^{8}$ = 256 ROs in each PUF instance. The average oscillation frequency is 101 KHz. The most important figures of merits for the PUF, namely, the uniqueness, reliability, power and security are presented and discussed in the following subsections.

### 3.1. Uniqueness

The uniqueness measures how different the CRPs produced by a PUF are from the other chips. The average inter-die Hamming Distance (HD) of the PUF’s CRPs is generally used to calculate the uniqueness. With the same input challenge *C* to two different chips, *u* and *v*, two *n*-bit responses $R_{u}$ and $R_{v}$ can be generated. The average inter-die HD for *m* chips is expressed as [17]:

(10) $$U=\frac{2}{m(m-1)}\sum_{u=1}^{m-1}\sum_{v=u+1}^{m}\frac{HD(R_{u},R_{v})}{n}\times 100\%$$

**Figure 7:**
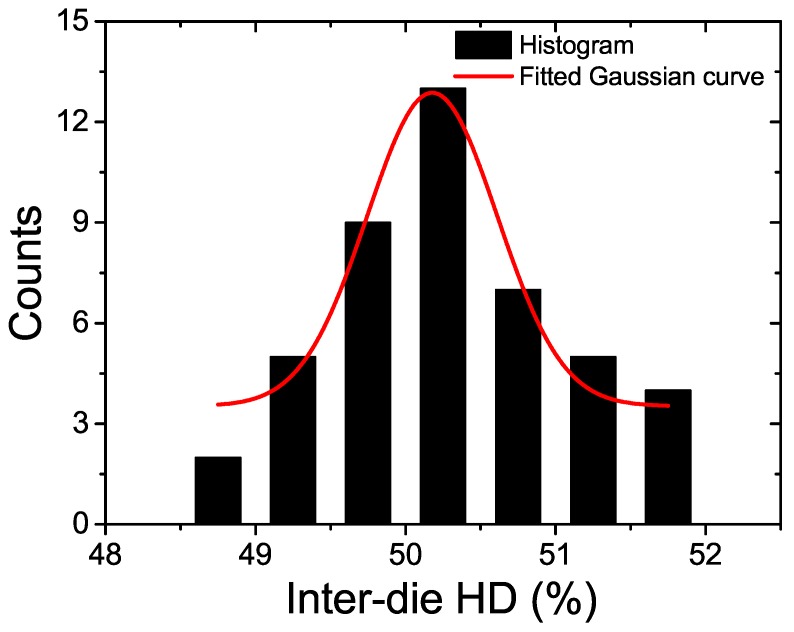
The measured inter-die HD distribution for the proposed RO PUF.

Figure 7 shows the measured frequency distribution of the inter-die Hamming Distance (HD) for 10 PUF dies. The uniqueness measured from the proposed PUF’s inter-die HD is 50.17%, with the ideal value equal to 50%. A Gaussian distribution with mean μ = 50.17% and standard deviation σ = 0.43% can well fit this distribution as shown in Figure 7.

### 3.2. Reliability

**Figure 8:**
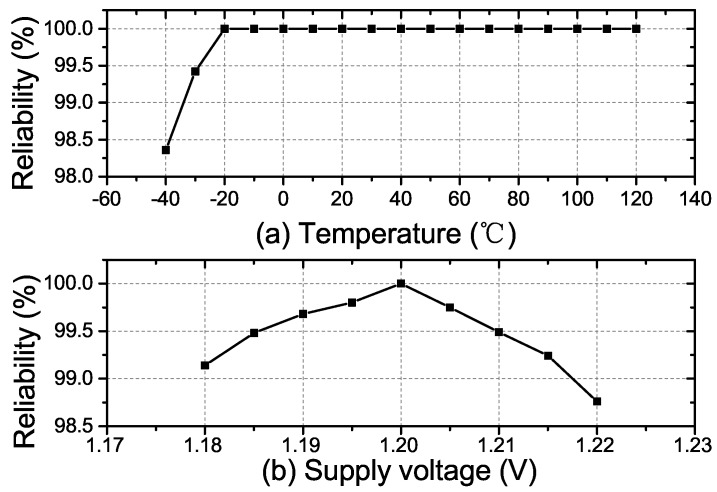
(**a**) The measured average reliability of the proposed RO PUF versus the temperature variations; (**b**) the measured average reliability of the proposed RO PUF versus the voltage variations.

The reliability represents how reproducible the CRPs generated by a PUF are, under variable operating conditions, e.g., different temperature, supply voltage, ambient noises, etc. The intra-die HD is a way to estimate the reliability. Given an input challenge *C*, each chip *i* produces an *n*-bit reference response $R_{i}$ under the normal operating condition. It is then measured *k* times with the same set of challenges under varying operation environments. The produced responses are $R_{i,j(j=1,2,\ldots ,k)}$. The assessing of reliability for chip *i* can be written as [17]:

(11) $$S=1-BER=1-\frac{1}{k}\sum_{j=1}^{k}\frac{HD(R_{i},R_{i,j})}{n}\times 100\%$$

where BER stands for the bit error rate. With 1000 CRPs produced by the PUF under various temperatures and supply voltages, the reliability is measured. Figure 8a,b show the fabricated RO PUF’s reliability with the temperature and voltage variations, respectively. The worst-case reliability is observed to be 98.30% at −40 ${}^{\circ }$C and 1.22 V. The proposed PUF is characterized with a high temperature reliability over a long temperature range. This is because the delay cell we implemented in each RO is the current starved inverter. The oscillation frequency of the RO using the current starved inverter is much less sensitive to the temperature variations [18].

### 3.3. Power Consumption

**Figure 9:**
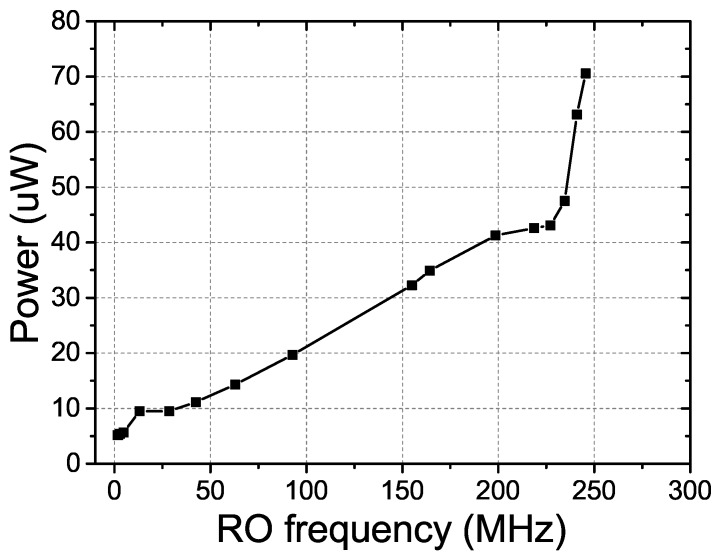
The measured average power consumption per challenge-response pair (CRP) of the proposed PUF at different ROs frequencies.

In our proposed RO PUF, the RO’s frequency and the power consumption can be optimized by tuning $V_{p}$ and $V_{n}$ of the current starved inverters. Figure 9 presents the measured average power consumption versus the RO’s frequency. It is indicated that the minimum measurable oscillation frequency is 100 KHz and the corresponding power consumption is only 5.16 μW. This low power consumption feature is more suitable for the resource-constrained WSN devices working in the distributed area.

### 3.4. Security Analysis

#### 3.4.1. SVM Attack

**Figure 10:**
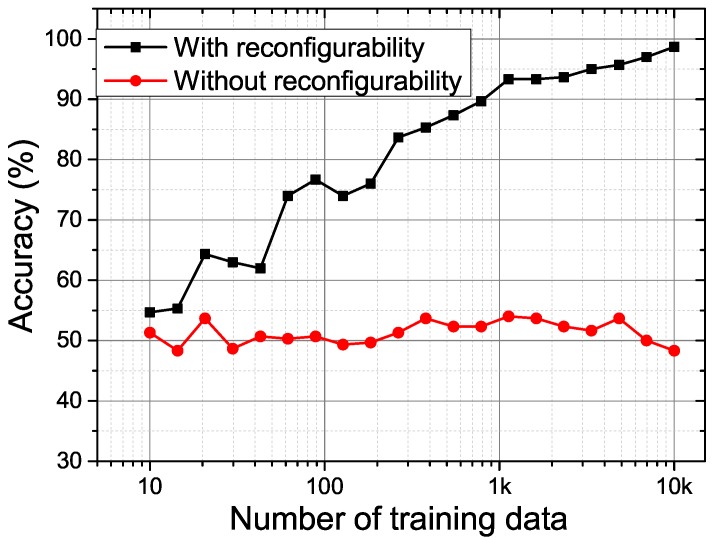
The prediction accuracy by support vector machine (SVM) for the proposed PUF with and without reconfigurability.

With the above-mentioned reconfigurability in Section 2, the proposed PUF is able to thwart the SVM modelling attack. In order to have a fair comparison with SVM attack on the 64-stage arbiter [11], we have extensively simulated a 64-stage proposed PUF using the UMC 65 nm CMOS technology to generate the enough training and testing CRPs. Figure 10 shows the prediction results for the proposed 64-bit RO PUF with and without reconfigurability using the tool $SVM^{light}$ [19]. The reconfigurability is disabled by fixing the value of $N_{clk}$ in the LFSR counter. The prediction accuracy is higher than 90% with only 1000 training CRPs for the RO PUF without reconfigurability. This is because, if the reconfigurable parameter $N_{clk}$ is fixed, the relation of *C* and $C^{\prime }$ is fixed. The model for the proposed PUF is similar to the arbiter PUF [11], which is very vulnerable to the SVM attack. However, when the reconfigurability is enabled by setting $N_{clk}$ as a random number, our proposed PUF is proved to be more resilient to the SVM modeling attack, as the SVM predication accuracy for our proposed PUF response only fluctuates around 50% even with a large training set size of ten thousand CRPs.

#### 3.4.2. EM Side-Channel Attack

EM side-channel attack has been successfully adopted to break the RO PUFs [12]. The analysis is based on the study and comparison of the detectable EM emanations’ frequency spectrum for the active RO. According to Friis transmission equation [20]:

(12) $$P_{r}=G_{t}G_{r}{\left(\frac{\lambda }{4\pi R}\right)}^{2}P_{t}$$

where $G_{t}$ and $G_{r}$ are the antenna gains of the transmitting and receiving antennas, respectively, λ is the wavelength, *R* is the distance from the detector probe to the device, $P_{r}$ is detectable magnitude of the EM radiation and $P_{t}$ is the device’s working power consumption. Our proposed hybrid RO PUF has lower power consumption over the classic RO PUF. Since $P_{r}$ is proportional to $P_{t}$, it will have a lower magnitude of EM radiation.

**Figure 11:**
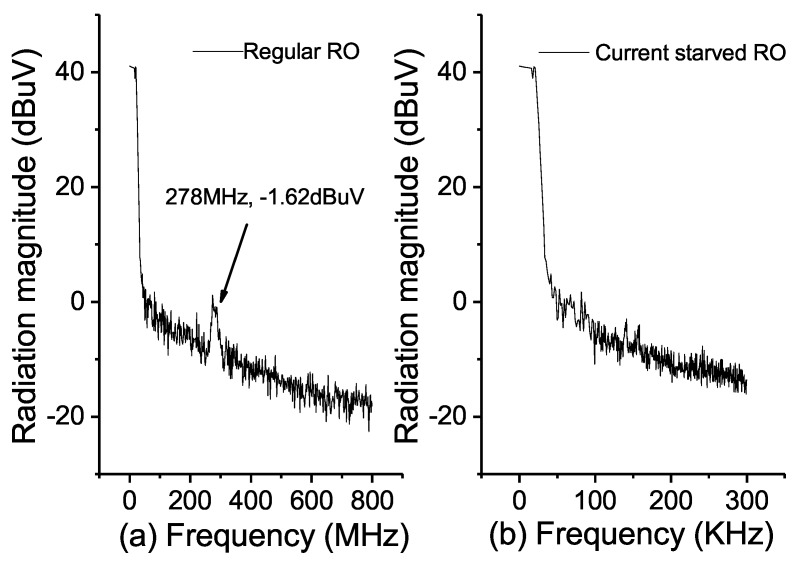
(**a**) The measured EM radiation for the regular RO; (**b**) the measured EM radiation for the proposed RO.

To validate our proposed PUF’s resilience against EM side-channel attack, *FLS 106* IC scanner is used to capture the EM radiation close to the fabricated chip’s surface. Figure 11a,b illustrate the measured spectrum of the regular RO and the proposed RO fabricated in the same chip, respectively. It is noticed that an EM radiation of 100.38 dBμV at 278 MHz is detected for the regular RO, however, the EM radiation magnitude of the proposed RO is too small to be distinguished from the noise floor. Additionally, as the proposed RO PUF only occupies a tiny silicon area with an interleaving structure, it is quite challenging to pinpoint it on the chip, which further elevated the proposed RO’s security performance [21].

## 4. Conclusions

In this paper, we present a compact RO PUF with high resilience to both the EM side-channel attack and the SVM modelling attack. By utilizing the current starved inverters as the RO’s delay cell, both the oscillation power and the emitted EM signal are minimized, leading to significantly enhanced immunity to the EM based side-channel attack. Additionally, the prototype of the proposed PUF fabricated using 65 nm CMOS process only consumes a low power of 5.16 μW per CRP at 1.2 V, under an oscillation frequency of 100 KHz. In addition, the measured CRPs exhibits a superior uniqueness of 50.17% and a BER of 1.7% with the operation temperature varied from −40 ${}^{\circ }$C to 120 ${}^{\circ }$C. Furthermore, with the external challenge randomized by the incorporated LFSR counter, the reconfigurable CRPs are capable of providing substantial resilience to the prediction attack by SVM. The proposed PUF shows great promise to a wide range of light-weight WSN security applications with limited battery capacity.
